# Applications of decellularized extracellular matrix in bone and cartilage tissue engineering

**DOI:** 10.1002/btm2.10110

**Published:** 2018-10-26

**Authors:** Yu Seon Kim, Marjan Majid, Anthony J. Melchiorri, Antonios G. Mikos

**Affiliations:** ^1^ Dept. of Bioengineering Rice University Houston TX 77005; ^2^ Biomaterials Lab Rice University Houston TX 77005

**Keywords:** bioink, bone, cartilage, decellularization, extracellular matrix, hydrogels, particles, scaffold

## Abstract

Regenerative therapies for bone and cartilage injuries are currently unable to replicate the complex microenvironment of native tissue. There are many tissue engineering approaches attempting to address this issue through the use of synthetic materials. Although synthetic materials can be modified to simulate the mechanical and biochemical properties of the cell microenvironment, they do not mimic in full the multitude of interactions that take place within tissue. Decellularized extracellular matrix (dECM) has been established as a biomaterial that preserves a tissue's native environment, promotes cell proliferation, and provides cues for cell differentiation. The potential of dECM as a therapeutic agent is rising, but there are many limitations of dECM restricting its use. This review discusses the recent progress in the utilization of bone and cartilage dECM through applications as scaffolds, particles, and supplementary factors in bone and cartilage tissue engineering.

## 1 INTRODUCTION

Regenerative medicine offers the ability to repair injuries that the body fails to heal. Although there are many synthetically designed materials to support tissue regeneration, these materials fall short of fully replicating a tissue's microenvironment.^1^ Looking to the function of this microenvironment for inspiration has provided insight into how materials used in tissue regeneration can be improved. One potential therapeutic material is the native extracellular matrix (ECM), which is the noncellular component of tissue that provides the structural support and biochemical cues for determining a cell's fate.^2^ ECM is a natural material that encompasses both the cell microenvironment and biochemical factors for living cells.^3^, ^4^ Each tissue type has a specialized ECM structure and composition that modulates cell responses and benefits the survival of cells within that tissue.^2^ ECM is composed of two major components, collagen and proteoglycans, which are secreted by cells and assembled in a manner specific to individual tissue types. It contains a reservoir of growth factors and cytokines; these send signals that regulate cell proliferation and migration as well as modulate differentiation and phenotypic expression of the cell. Due to its inherent compositional similarity and modulatory abilities of supporting tissue growth and differentiation, the use of tissue‐specific ECM for tissue regeneration has gained popularity, including in the areas of bone and cartilage engineering.

Bone ECM consists of an organic and inorganic phase. The organic phase, mostly type I collagen, provides the tissue with flexibility, while the inorganic phase, mainly consisting of calcium phosphate, specifically hydroxyapatite (HA),^5^ is the source of bone strength.^6^ In addition, there are four cell types in bone tissue that contribute to osteogenesis: (a) undifferentiated osteoprogenitor cells, (b) matrix‐depositing osteoblasts, (c) mature osteocytes that no longer deposit matrix, and (d) osteoclasts that resorb bone tissue. In natural maintenance of the tissue as well as in response to injury, these cell types work in conjunction to homeostatically build up and breakdown the matrix.^7^ Bone tissue is one of the few tissues that can heal itself with little to no formation of scar tissue. However, there is a critical size limit of 2.5 cm for most bone,^8^ above which regeneration will not occur. In these cases, it is necessary to induce and support osteogenesis to heal the defect.

Cartilage ECM is primarily a collagenous network,^3^ with varying compositions and types of collagen depending on the cartilage type. Hyaline cartilage is mainly type II collagen, while fibrous cartilage is a mixture of both type I and II collagens.^9^, ^10^ Another major component of these networks is proteoglycans. Proteoglycans consist of multiple chains of glycosaminoglycans (GAGs) branching off from a core protein. Aggrecan is the most abundant proteoglycan present in cartilage, of which chondroitin sulfate and keratan sulfate are the main GAG components. Aggrecan is highly anionic at physiological pH and attracts water molecules, which gives cartilage its elastic and swelling properties, allowing it to have high shock absorbance under compressive load.^3^, ^11^ Different cartilage types have varying collagen/GAG compositions, giving each type distinct mechanical properties. For instance, knee meniscus, a type of fibrocartilage, is predominantly composed of type I collagen, but it has lower GAG content compared to hyaline cartilages such as articular cartilage. This results in meniscus having a higher tensile modulus and lower compressive modulus compared to articular cartilage.^12^ Cartilage ECM is maintained solely by chondrocytes, which comprise only 1‐5% of total cartilage volume.^13^ This low cell density contributes to cartilage tissue having low regeneration capabilities, which is also compounded by the avascular nature of the tissue. In cases when healing does occur, it often yields the formation of fibrous cartilage, which leads to stiffer tissue at the injury site and long‐term performance issues.^3^, ^11^ To improve function, regenerative therapies promote the formation of native articular/hyaline cartilage rather than fibrous cartilage.

To process ECM for use in regenerative therapy, the excised tissue must first undergo decellularization. Decellularization refers to the process of treating a tissue with any combination of physical stress and chemical/enzymatic agents to remove cellular components, leaving behind only the noncellular ECM that can be used for therapeutic applications. The specific method of decellularization used depends on the tissue type; for instance, while cartilage tissue is able to undergo a relatively harsh treatment, lung tissue requires a more sensitive decellularization method to preserve its tissue composition.^14^ The resulting decellularized extracellular matrix (dECM) can then be processed further for different tissue engineering applications. These applications are summarized in Table 1. The main benefit of dECM is that it retains components of the natural cell environment^2^; with proper decellularization, the complex biomolecular and physical cues in the ECM are preserved and can support cell growth and viability. Unlike in transplanted tissue, dECM has a lower risk for immune response because almost all the cellular DNA is removed.^15^ However, the decellularization process does present challenges, the foremost of which is maximizing the removal of cellular material while limiting damage to the ECM.^15^

Although synthetic materials have their benefits, such as tunability of physicochemical properties, they are unable to fully replicate the native microenvironment and structure of the tissue, even with modifications or the addition of bioactive factors.^1^ Thus, incorporating dECM presents a promising method for creating an environment that better mimics that of native tissue and suits repair of the injury site.

## 2 GENERAL METHODS OF DECELLULARIZATION

To retain as much of the tissue's bioactivity as possible while maximizing the removal of nuclear material, the decellularization process must minimize the loss of native ECM components. Implantation of decellularized tissue that has had its nucleic materials incompletely removed or degraded could result in host foreign body reaction, which leads to the formation of fibrous capsule surrounding the implant site.^16^, ^17^ This eventually can result in improper tissue remodeling and therefore limit the regenerative potential of the decellularized tissue.^18^ Preserving the ECM ultrastructure is also important in applications where dECM is not further processed but used as a scaffold by itself. Specific decellularization procedures vary according to the tissue type and can involve a combination of (a) physical, (b) enzymatic, and (c) chemical processes. The most frequently used techniques are discussed below.

#### 2.1. Physical decellularization

Introducing physical stresses such as freeze–thaw and osmotic pressure can result in cell lysis without significantly disrupting the ultrastructure of the tissue. Freeze‐thawing is one of the most widely used physical decellularization methods, during which the formation of ice crystals puncture cell membranes. The cycle is repeated multiple times before the tissue sample can be processed further. Another option is osmotic lysis, during which tissues are placed in either a hypertonic^19^ or hypotonic solution such as deionized water^20^ that ruptures the plasma membrane via osmotic shock. Other common physical decellularization methods include hydrostatic pressure,^21^ sonication,^22^ and electroporation.^23^

Tissues that undergo only physical decellularization, specifically freeze‐thawing, are considered to be *devitalized* but not decellularized, as the cells have been lysed, but the cell debris and genetic material still remain within the processed tissue. These samples are most often processed into particles, during which the tissues go through a combination of freeze‐thawing and lyophilization, and are then ground into powder using a freezer/mill.^19^, ^24^, ^25^, ^26^, ^27^ Devitalized tissue particles have been shown to have higher quantities of ECM components, such as GAGs, than those that have been additionally treated with chemical/enzymatic decellularization methods.^19^, ^25^ However, there are safety concerns of possible immune responses that could result from residual cellular material. There have also been conflicting reports on whether the increase in ECM components leads to a better cellular response.^19^ For instance, rat bone marrow‐derived mesenchymal stem cells (MSCs) cultured with devitalized cartilage (DVC) particles had lower cell viability and chondrogenic gene expression levels than MSCs cultured with decellularized cartilage (DCC) particles.^19^ This difference may be due to the increased quantity of GAGs in devitalized tissue causing the particles to be too dense for adequate cell infiltration.^19^

Physical decellularization is the least disruptive decellularization method, with most of the ECM components and structure left intact after treatment.^28^ However, physical decellularization alone cannot completely remove cellular debris from the tissue. Often it is used in conjunction with additional chemical or enzymatic methods.^29^ Similarly, incubating a tissue sample in chemical or enzymatic agents without physical agitation does not result in an acceptable degree of decellularization due to limited diffusion into the tissue. Therefore, a combination of all three methods has a synergistic effect where physical agitation enhances the tissue penetration depth of chemical and enzymatic agents, thereby facilitating the removal of lysed cell material.^30^, ^31^

#### 2.2 Chemical decellularization

Chemical methods of decellularization can largely be divided into two subcategories where tissue samples can be treated with either (a) acidic or basic conditions or (b) detergents. Treating tissues with acids or bases results in cell degradation and the removal of cellular components such as nucleic acids. The degree of successful decellularization will vary according to the type and concentration of the acid/base being used, processing time, and the type of tissue being treated. Bases are considered the harsher option of the two and can result in significant loss of GAGs.^32^, ^33^ Preservation of GAGs during decellularization is important to maintain tissue mechanical properties (e.g.,tensile, viscoelastic properties)^33^, ^34^ and to retain growth factors in the tissue,^35^, ^36^ the latter of which have been linked with enhanced biocompatibility in vitro.^37^ Except for cases where reduction of GAGs is a desired outcome,^20^ alkaline treatment is rarely used as an option for decellularization of bone and cartilage tissue. Peracetic acid is frequently used to decellularize thin tissues such as small intestine submucosa (SIS). For more dense tissues such as menisci, formic acid is considered the best choice for removing both collagen and GAGs.^38^

Chemical decellularization can also be performed through the use of detergents. Three main types of detergents are used: nonionic, ionic, and zwitterionic. Nonionic detergents such as Triton X‐100 lyse cells through insertion into the lipid bilayer, disrupting the cellular membrane. While disrupting lipid interactions, these largely preserve protein–protein interactions.^39^ Proteins are solubilized, but their native structure mostly remains intact.^18^ Ionic detergents such as sodium dodecyl sulfate (SDS) are known as denaturing detergents; they disrupt cell membranes and also completely denature proteins. Generally, ionic detergents are considered harsher than nonionic detergents, and they are more detrimental to the ECM structure. For instance, MSCs seeded on tendons treated with SDS had a lower viability and distribution throughout the tissue than cells seeded on tissues treated with Triton X‐100.^40^ Zwitterionic detergents such as 3‐([3‐cholamidopropyl] dimethylammonio)‐1‐propanesulfonate (CHAPS) have a net zero charge and show characteristics of both ionic and nonionic detergents.^15^ Although zwitterionic detergents result in less denaturing of proteins compared to ionic detergents, they also tend to remove less cellular material than ionic detergents.^41^, ^42^

#### 2.3 Enzymatic decellularization

Enzymatic decellularization is most often used directly after chemical decellularization to further facilitate the cell degradation and the removal of residual nuclear material from the tissue. Nucleases and proteases are the most widely used enzymes for enzymatic decellularization. Nucleases, such as deoxyribonuclease and ribonuclease, act directly on DNA and RNA chains, respectively, to hydrolyze phosphodiester bonds. Proteases, such as trypsin, act on proteins by hydrolyzing peptide bonds. Trypsin is a serine protease that cleaves the carbonyl side of lysine or arginine residues. Because of its specific activity on peptides, trypsin treatment can severely disrupt ECM proteins such as elastin and collagen.^15^ Enzymatic methods are frequently used in conjunction with chelating agents such as ethylenediaminetetraacetic acid (EDTA), which disrupt cell adhesion to ECM proteins by sequestering metallic ions such as calcium.^15^

#### 2.4 Evaluation of the degree of decellularization

Measuring the amount of double‐stranded DNA (dsDNA) in dECM is the current gold standard for evaluating the degree of successful decellularization. By comparing the amount of DNA in tissue samples before and after decellularization using quantitative assays such as PicoGreen,^43^ it is possible to make general conclusions on whether the sample has been sufficiently decellularized. Crapo et al. have suggested three minimum criteria that tissues should satisfy to be considered successfully decellularized: (a) tissue samples should contain < 50 ng of dsDNA per mg of dry ECM, (b) any remaining DNA fragments should be smaller than 200 base pairs, and (c) the tissue should not have visible nuclear material when stained with DAPI or hematoxylin & eosin.^15^ In addition to quantifying the remaining cellular material, it is also important to evaluate both the macroscopic change in the ECM structure and the biochemical composition to ensure minimal disruption of the ECM composition. For applications where the whole tissue is decellularized without being further broken down into smaller particles, imaging techniques such as scanning/transmission electron microscopy^40^, ^44^ and microcomputed tomography^45^, ^46^ can be used to compare the structure of ECM both before and after decellularization. For more quantitative analyses, the amount of sulfated glycosaminoglycans (sGAGs) and collagen can be evaluated using dimethylmethylene blue (DMMB)^47^ and hydroxyproline assays,^48^ respectively. These assays are well‐established and can help investigators draw more concrete conclusions about the efficacy of the decellularization protocol used.

## 3 POSTDECELLULARIZATION PROCESSING METHODS

### 3.1 Decellularized ECM as a scaffold

One of the simplest methods of using dECM is as a scaffold that maintains its original geometry. The biggest advantage of this method is that, compared to other processing methods that completely pulverize the dECM, using it as an unprocessed scaffold suggests that the tissue retains a large portion of its original ECM architecture. dECM can be prepared from various tissue types to accommodate different compositions, topographies, and mechanical properties.^49^ However, such benefits can only be obtained if (a) most of the cell debris is removed from the tissue without destruction of essential ECM components such as GAGs and collagen fibers and (b) the dECM can be thoroughly recellularized. As a result, recent literature has focused on the effects of decellularization methods on the composition and ultrastructure of the resulting tissue and the degree of recellularization.

#### 3.1.1 Bone

The development of a decellularized bone scaffold has been motivated by the need to improve the biocompatibility of allograft bone^50^ and the benefit of preserving the bone's native structure.^45^, ^46^ Xu et al. successfully decellularized annulus fibrosus tissue from porcine spine while maintaining its macroscopic structure.^45^ Different types of decellularization methods showed various effects on the retention of ECM molecules; trypsin treatment resulted in lowest GAG content, followed by SDS and Triton X‐100. All three methods did not result in significant loss of collagen.^45^ Smith et al. investigated the effect of donor age on the resulting osteogenic capacity of the isolated human bone following decellularization.^46^ The authors reported that bone from an old donor (≥70 years age) was more porous and less dense than that from a young donor (≤50 years age), but the tissues otherwise had similar composition (e.g.,mineral density, calcium/phosphate ratio). MSCs seeded on decellularized bones from older donors expressed higher levels of osteogenic markers than those seeded on decellularized bones from young donors, which the authors attributed to enhanced porosity. Decellularized bone has also been subjected to further modifications, such as collagen/HA coating.^51^ When type I collagen solutions were applied to the surface of decellularized porcine cancellous bone, the coating modulated the stiffness of the matrix. Higher collagen concentration led to higher matrix stiffness compared to uncoated matrices, which in turn guided more robust differentiation of seeded MSCs into osteogenic lineages.

#### 3.1.2 Cartilage

Due to its dense ECM, decellularizing cartilage and seeding cells afterward have proven challenging. Multiple methods have thus been developed to improve the aforementioned processes, albeit with limited success. Luo et al. introduced channels into full‐thickness porcine cartilage discs, which acted as conduits for fluids and cells to penetrate into the tissue.^52^, ^53^ These channels supported cell viability and attachment while also allowing the cells to align with the native collagen architecture. However, the degree of recellularization throughout the cartilage tissue was still limited compared to native tissue, indicating that the formation of physical channels was not enough to allow sufficient recellularization. After reports claiming that proteoglycans in cartilage inhibited cell adhesion,^54^, ^55^ there have also been attempts to enhance cell adhesion following decellularization by removing GAGs from cartilage tissue.^56^, ^57^, ^58^, ^59^ Bautista et al. added chondroitinase ABC during decellularization to aid the removal of GAGs from porcine articular cartilage.^60^ The authors also created channels through the tissue, which was then overlaid with cell suspension and centrifuged (to pull the cells deeper into the tissue). These treatments, although successful in enhancing decellularization, did not improve recellularization rates. Tyler et al. conducted an in vivo study using the ovine osteochondral defect model, during which a decellularized osteochondral allograft was implanted and studied after 12 weeks.^61^ The constructs were remodeled by infiltrating cells, but the cell density was still lower than that of healthy cartilage, resulting in a low GAG concentration within the decellularized implant.

Although maintaining the native architecture during decellularization has its benefits, dECM scaffolds are limited to certain geometries and cannot easily be scaled. To circumvent this, decellularized tissue can be pulverized and freeze‐dried into particles that are packed into molds, making it possible to fabricate highly porous dECM scaffolds of varying geometry.^59^, ^62^, ^63^, ^64^, ^65^ Architectural attributes like the size of pores within these scaffolds can impact the behavior of seeded cells. Almeida et al. prepared coarse and fine dECM particles by processing porcine cartilage using two different methods (homogenizer and cryomill, respectively).^59^ By changing the concentration of dECM particle slurry, the authors were able to prepare scaffolds with pore sizes ranging from 32 ±12 to 65 ±20 μm. Scaffolds with larger pore sizes resulted in better cell infiltration, proliferation and higher chondrogenic activity in vitro. To maintain structural fidelity and resolution of architectural features, these scaffolds usually undergo physical crosslinking such as UV irradiation or dehydrothermal treatment (DHT).^36^, ^66^ Gawlitta et al. prepared porous scaffolds from decellularized equine cartilage and crosslinked them via UV irradiation.^63^ The scaffolds were seeded with MSCs and were subcutaneously implanted in immunocompromised rats to measure osteoinductive capacity. MSCs within the dECM‐based porous scaffolds experienced chondrogenic differentiation which allowed for enhanced endochondral bone formation.^63^

The behavior of cells and physical properties of the scaffolds can be affected by different crosslinking schemes.^62^, ^67^ Rowland et al. reported that physical crosslinking, specifically DHT crosslinking, resulted in better chondrogenic response from encapsulated MSCs than did chemical crosslinking.^62^ However, a study conducted by Almeida et al. showed chemical crosslinking did not limit the chondroinductive capacity when compared to a scaffold crosslinked via DHT.^59^ These contradictory results indicate that further studies must be conducted to fully understand the impact of either physical or chemical crosslinking on dECM‐based porous scaffolds and their ability to induce differentiation in stem cells.

### 3.2 Solubilized dECM as a hydrogel

Solubilized dECM is created when dECM is further digested using pepsin, creating a homogeneous solution that can undergo thermal gelation at physiological temperature and pH. As the tissue is homogenized, solubilized dECM does not preserve either the architecture or topology of the natural ECM. One of the earliest attempts to create such tissue‐derived hydrogels was made with decellularized SIS.^68^ The tissue, following decellularization, was pulverized in liquid nitrogen, and the resulting powder was digested in an acidic buffer containing pepsin. Digested SIS demonstrated thermally responsive gelation by maintaining the pH and ionic strength of the solution at a physiologically relevant level and placing the solution in a mold at 37 °C for 30 min to an hour.

The method of fabricating a hydrogel using solubilized dECM has remained similar over the course of its use and is as follows: (a) tissue decellularization, (b) digestion with pepsin in acidic buffer, (c) neutralization of the buffer to physiological pH/salt concentration using 10 × phosphate buffered saline (PBS) and NaOH, and (d) formation of the hydrogel by bringing the temperature to 37 °C. Although pepsin digestion is the most widely used method for solubilizing dECM, the bioactivity of pepsin‐digested dECM remains controversial.^69^, ^70^ To this end, several studies have explored the possibility of using urea to extract soluble components of dECM.^71^, ^72^ Urea is a chaotropic agent that disrupts hydrogen bonding, resulting in the denaturation of proteins and the disruption of interactions between lipids and proteins. Urea‐extracted dECM had higher concentrations of small and moderate MW proteins compared to pepsin‐digested dECM, which consisted primarily of collagen chains.^71^ When used as a supplement in two‐dimensional/three‐dimensional (3D) cell culture, urea‐extracted dECM also promoted tissue‐specific differentiation of MSCs.^72^ Chondrogenic activity was upregulated when the urea‐extracted dECM was mixed with gelatin methacrylate (GelMA) hydrogels, although the effect was short term.^72^

#### 3.2.1 Bone

**Figure 1:**
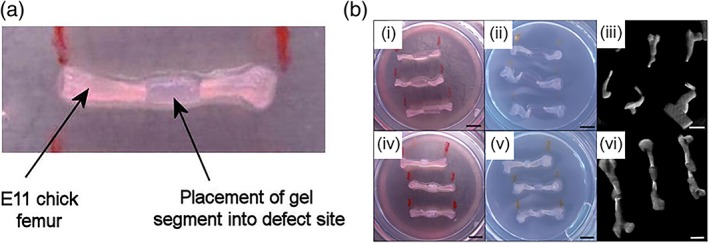
Examining the regenerative potential of solubilized bone dECM hydrogel using ex vivo model. (a) hydrogel was loaded within the 2 mm defect site created in an embryonic Day 11 chick femur. (b) Alginate hydrogel was not incorporated into the defect site after 10 days of culture (i–ii), however alginate combined with solubilized bone dECM maintained its geometry for 10 days (iv–v). Micro‐computed tomography showed similar results (iii, vi). Reprinted from Ref. 78, Copyright 2014, with permission from Elsevier

The motivation for developing solubilized bone dECM comes from the inconsistent results obtained with delivering demineralized bone particles into bone defect sites using carriers such as sodium hyaluronate or gelatin.^73^, ^74^, ^75^ Sawkins et al. have demonstrated that both demineralized and decellularized bovine bone ECM, after undergoing pepsin digestion, could form a thermally responsive hydrogel.^76^ Both demineralized and decellularized bone ECM consisted primarily of randomly oriented type I collagen fibers and could support the growth of mouse primary calvarial cells. Cells encapsulated in dECM hydrogels showed a higher proliferation rate than those in demineralized bone or collagen type I hydrogels. Paduano et al. demonstrated that dental pulp stem cells (DPSCs), when seeded on dECM derived from bovine tibia, underwent odontogenic differentiation in vitro.^77^ Adding exogenous growth factors such as basic fibroblast growth factor (bFGF) and epidermal growth factor (EGF) to dECM hydrogels had a synergistic effect on the degree of differentiation.^77^ In a series of studies published in 2014, Smith et al. used solubilized bovine bone dECM mixed with alginate as a carrier hydrogel to deliver cells and growth factor‐releasing microparticles to support the regeneration of skeletal tissue.^78^, ^79^ Solubilized dECM was injected between the segmental defect of embryonic Day 11 chick femur and cultured ex vivo for 10 days after which the degree of bone tissue formation was evaluated. Alginate/solubilized dECM hydrogels incorporating human bone marrow stem cells (BMSCs) and osteogenic growth factor‐loaded poly(lactic‐*co*‐glycolic acid) (PLGA) microparticles induced higher collagen deposition, indicating osteoid matrix deposition (Figure 1).^78^ With its inherent ability to undergo thermogelation, solubilized dECM is a versatile platform that can be combined with other materials to form a potent and tunable hydrogel capable of inducing bone regeneration through effective cell growth and differentiation.

**Table 1:** Advantages and disadvantages of different postdecellularization processing methods

Application	Specific method (following decellularization)	Advantages	Disadvantages	References
Scaffold				
• Whole tissue	dECM stored until further use	Ease of fabrication	Difficulty with decellularizing and recellularizing dense tissues	45, 46, 49, 50, 51, 52, 53, 54, 55, 56, 57, 58, 60, 61
• Molding	Homogenized into powder and freeze‐dried in molds	Adjustable scaffold geometry	Poor mechanical properties compared to whole tissue scaffold	59, 62, 63, 64, 65, 67
Solubilized dECM as a hydrogel				
	Powdered dECM digested with pepsin, or extracted with urea	Thermosensitive gelation at physiological temperature	Disruption of native architecture of the dECM during digestion/extraction	26, 62, 68, 69, 70, 71, 72, 73, 74, 75, 76, 77, 78, 79, 80, 81, 82, 83, 84
dECM particle		Cell signaling cues provided to synthetic materials that are prepared using various methods	Negative impact on physical properties of the synthetic scaffold	
• Hydrogel incorporation	dECM particles incorporated into hydrogel during formation			111, 112, 113
• 3D‐ornamented printing	dECM particles mixed with polymer, which is then melted and extruded			107, 115, 122
• Electrospinning	dECM particles dissolved with polymer and electrospun			27, 114
Bioink				
	Powdered dECM digested in pepsin in acidic buffer, then pH adjusted using NaOH dECM pre‐gel solution used as bioink	Fabrication of complex architecture scaffolds with multiple materials	Often difficult to print without modification	99, 101, 102, 103
Cell‐laid matrix				
	Cells seeded on polymeric scaffold or surface where ECM could be deposited; scaffold/surface subsequently decellularized	Even coating of ECM on complex surfaces	In vitro generated ECM cannot reproduce that of the native tissue	4, 85, 86, 87, 88, 89, 90, 91, 92, 93, 94, 95, 96, 97, 98

*Note*. dECM=decellularized extracellular matrix; 3D=three‐dimensional; ECM=extracellular matrix.

#### 3.2.2 Cartilage

Wu et al. were able to fabricate a thermoresponsive hydrogel from porcine menisci dECM, which could support the growth of both seeded and encapsulated chondrocytes in vitro.^80^ Digested dECM solutions were subcutaneously injected into mice and gelled within 30 minutes. The resulting hydrogel showed good cytocompatibility without causing an inflammatory reaction in vivo. Kwon et al. have also shown that it is possible to use articular cartilage‐derived dECM as an injectable drug delivery vehicle.^81^ In this study, the hydrogel was injected subcutaneously into rats and showed sustained release of bovine serum albumin over 10 days in vivo. Like solubilized bone dECM, cartilage solubilized dECM can be used to deliver cells, drugs, and other bioactive molecules for cartilage regeneration.

The most common limitation of hydrogels formed from solubilized dECM is poor mechanical properties. For this reason, different approaches to enhance the mechanical properties of solubilized dECM have been implemented, including mixing with biological polymers, such as alginate,^78^, ^79^ and crosslinking using physical, UV photochemical,^26^, ^62^, ^82^ or chemical^83^, ^84^ methods. Although such treatments create hydrogels that are physically stable, they can also limit the chondrogenic capacity of the hydrogel. For instance, Cheng et al. showed that although crosslinking the hydrogel using genipin prevented cell‐mediated contraction, increasing the crosslinking density reduced cell infiltration and chondrogenic activity, possibly due to a decrease in pore size and a reduction in adhesion sites.^83^ This can be overcome through combinatorial material techniques, including the addition of nonsolubilized dECM particles to a hydrogel. For example, Beck et al. showed that adding nonsolubilized dECM particles to a solubilized DVC hydrogel demonstrated enhancement of both the mechanical properties of a solubilized DVC hydrogel paste and chondrogenic gene expression from rat MSCs within the hydrogel both in vivo and in vitro.^26^

### 3.3 Cell‐laid matrix

ECM ornamentation or decoration is the process of coating a scaffold (often one derived from synthetic materials) with a layer of cell‐secreted ECM and is generally carried out in three steps: (a) cells are seeded onto a prefabricated synthetic or biological scaffold and cultured to promote ECM deposition, (b) the scaffold is decellularized, leaving behind the ECM‐ornamented scaffold, and (c) the ECM‐ornamented scaffold is recellularized for use as a regenerative therapy.^85^ The layer of dECM provides cell adhesion sites and biochemical cues that improve cell–material interactions compared to cell adhesion onto a bare scaffold. ECM ornamentation thus primes the scaffold for better cell attachment and more robust cell growth/proliferation.^85^ This process has been demonstrated on multiple types of scaffolds, such as 3D‐printed scaffolds,^4^, ^85^, ^86^ electrospun scaffolds,^87^, ^88^, ^89^, ^90^ and decellularized tissue.^91^, ^92^, ^93^

#### 3.3.1 Bone

**Figure 2:**
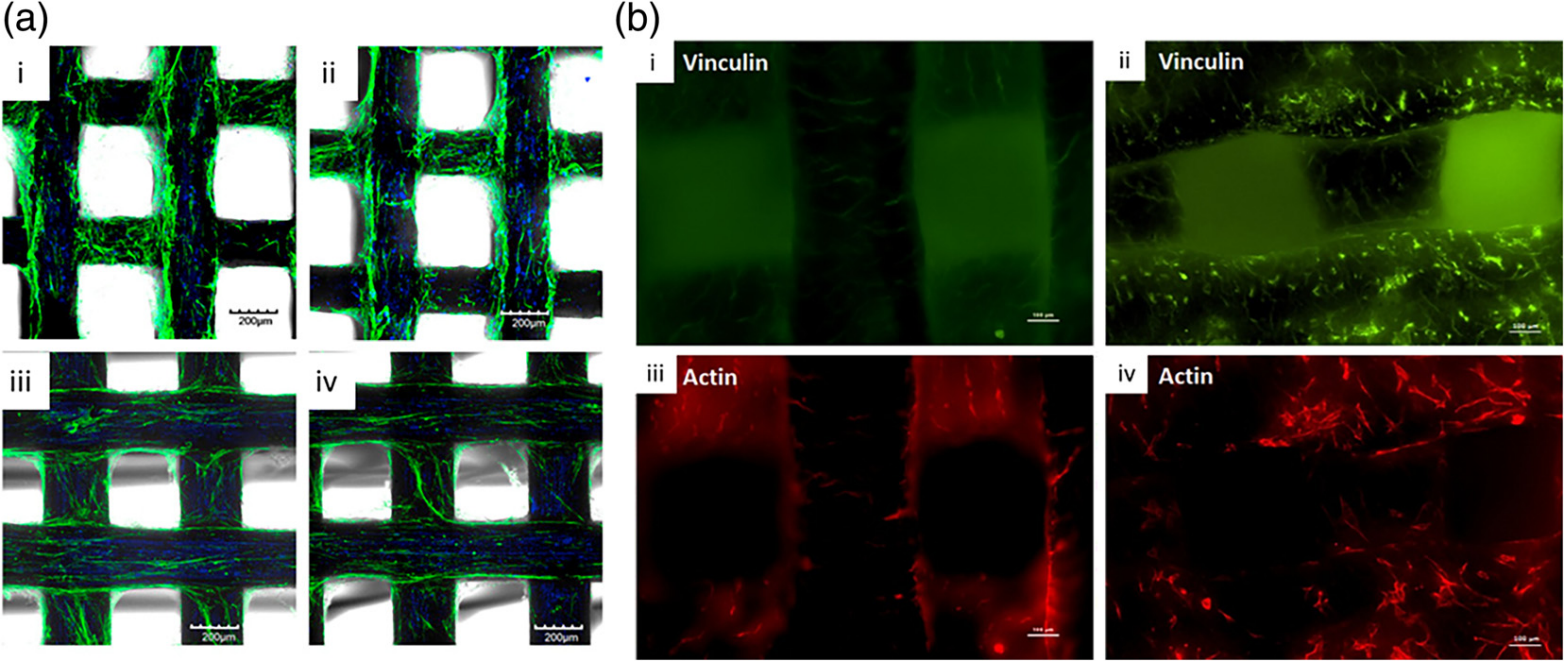
Cell‐laid matrix on 3D printed PCL scaffold. (a) Expression of F‐actin of hTMSCs on PCL/PLGA scaffolds with (i) addition of ECM/TCP, (ii) addition of ECM alone, (iii) addition of TCP alone, and (iv) no additions. (b) Fluorescent micrographs of protein expression for (i) vinculin on bare‐HA scaffold, (ii) vinculin on ECM‐ornamented scaffold, (iii) actin on bare‐HA scaffold, (iv) actin on ECM‐ornamented scaffold. (a) Reprinted from Ref. 4, Copyright 2014, with permission from Elsevier. (b) Reprinted with permission from Ref. 85. Copyright 2016 Wiley Periodicals Inc

Pati et al. performed an in vitro study where they 3D‐printed a scaffold of combined polycaprolactone (PCL), PLGA, and β‐tricalcium phosphate (TCP).^4^ The scaffold was seeded with human inferior turbinate tissue‐derived mesenchymal stromal cells (hTMSCs) to ornament it with cell‐laid bone ECM then decellularized to leave behind the ECM‐ornamented scaffold. After seeding with new hTMSCs, they showed that the presence of bone ECM significantly improved further deposition of mineralized matrix and enhanced osteogenic differentiation of the hTMSCs. They also performed an in vivo ectopic rat study with ECM‐ornamented scaffolds recellularized with human BMSCs. The ECM‐ornamented scaffold showed increased bone formation compared to the same scaffold with no ECM (Figure 2a).^4^ Kumar et al. 3D printed a PCL‐HA scaffold on which they seeded osteoblasts to deposit mineralized ECM.^85^ The scaffold was decellularized, leaving only the mineralized ECM, then reseeded with osteoblasts. The authors observed enhanced expression of the skeletal protein actin and the adhesion protein vinculin compared to undecorated scaffolds (Figure 2b). They also found improved cell–scaffold and cell–cell interactions, which correlate to increased cell adhesion, growth, and motility compared to bare PCL‐HA scaffolds.^85^ Electrospun scaffolds have also been used as templates for cell‐laid ECM.^87^, ^88^, ^90^ Thibault et al. seeded rat MSCs on electrospun PCL scaffolds and cultured them in well plates with osteogenic media for 12 days.^87^ The resulting ECM‐ornamented scaffolds triggered higher amount of calcium deposition from seeded MSCs when compared to bare PCL scaffolds, indicating that the presence of ECM coating enhanced the degree of osteogenic differentiation. To improve the distribution of ECM proteins throughout the thickness of electrospun PCL scaffolds, Liao et al. cultured MSC‐seeded scaffolds in flow perfusion bioreactors for up to 16 days.^90^ Longer duration of the culture resulted in better distribution and increased amount of ECM proteins and calcium, resulting in increased alkaline phosphatase activity and calcium deposition by seeded MSCs.

Aside from synthetic polymers, using decellularized SIS for ECM ornamentation has also been investigated.^93^, ^94^ Zhang et al. fabricated SIS ornamented with osteoblast‐secreted ECM that promoted both adhesion and proliferation of seeded adipose‐derived stem cells (ADSCs). Once implanted, the construct enhanced bone regeneration in vivo compared to non ECM‐ornamented or non ADSC‐seeded SIS.^94^

#### 3.3.2 Cartilage

For cartilage regeneration, the same coating techniques can be applied. However, there has also been substantial work in applying these techniques in tissue culture flasks to promote ECM deposition and study its effects in cell culture.^95^, ^96^, ^97^, ^98^ Hoshiba et al. cultured chondrocytes at different passages (0, 2, and 6) on tissue culture flasks for 10 days before decellularizing and collecting the ECM.^95^ ECM secreted by chondrocytes at different passages had different compositions, which impacted the behavior of chondrocytes that were seeded on dECM: cells seeded on P0 chondrocyte‐secreted ECM expressed higher levels of ACAN and COL2A1 (markers for aggrecan and type II collagen, respectively) than both uncoated and P2 and P6 chondrocyte‐secreted ECM groups, indicating that dECM limited the dedifferentiation of seeded chondrocytes.

ECM derived from these cell cultures can be used in translatable scaffolding strategies. Tang et al. cultured autologous bone marrow‐derived MSCs on tissue culture flasks, collected the cell‐deposited ECM, and freeze‐dried it to fabricate a porous cell‐derived scaffold.^97^, ^98^ Chondrocytes seeded on the dECM scaffold synthesized more GAGs and type II collagen than those seeded on atelocollagen (scaffold derived from bovine Achilles tendon fibers). A similar trend was observed in vivo, where dECM scaffold groups not only contained more GAGs, but also had a higher compressive modulus than the control group.^97^ The scaffold was also used with bone marrow stimulation (BMS) technique in a rabbit model, which further enhanced the degree of tissue regeneration compared to BMS only treatment.^98^

ECM ornamentation directly addresses many of the limitations of other tissue‐engineered dECM applications. As cells are seeded onto prefabricated scaffolds, complex geometries and tunable mechanical, physical, and biological properties can still be achieved through processes such as 3D printing of a synthetic scaffold while still utilizing the biological cues of natural ECM.^85^ It can limit or prevent the disadvantages of other techniques, such as the effect of high pressure extrusion on cells during 3D printing, the dimensional mismatch between defect site and fabricated scaffold in implantable hydrogels, and the lack of structural support in dECM alone.^4^ In addition, cell‐directed ECM‐ornamenting offers a more physiologically relevant microenvironment formation than a simple, manual coating with dECM, as coating can yield fragmented ECM components that do not accurately represent native ECM.^4^ Thus, by employing ECM ornamentation, scaffolds can be fabricated from tunable synthetic materials in complex geometric and synergistically incorporate natural ECM proteins to better recapitulate the ECM environment.^4^

### 3.4 Bioink

Another interesting application of dECM is its use as a 3D printable bioink. 3D printing, or additive manufacturing, is the creation of three‐dimensional structures layer‐by‐layer. The most common method of 3D printing bioinks is extrusion printing, during which the material is deposited via a mechanically controlled syringe into a desired geometry.^99^ This technique is capable of creating complex architectures by depositing multiple materials with high spatial control. These complex geometries can be obtained through the design of a computer model to match a defect site identified via medical imaging modalities such as MRI or CT.^99^ Bioactive molecules such as growth factors and different cell types can be incorporated into the scaffold to better mimic complex tissue architectures, such as the transition from bone to cartilage in an osteochondral defect.^100^ Porosity can also be tightly controlled within 3D printed constructs to ensure adequate gas and waste exchange as well as nutrient delivery.^100^

#### 3.4.1 Cartilage

**Figure 3:**
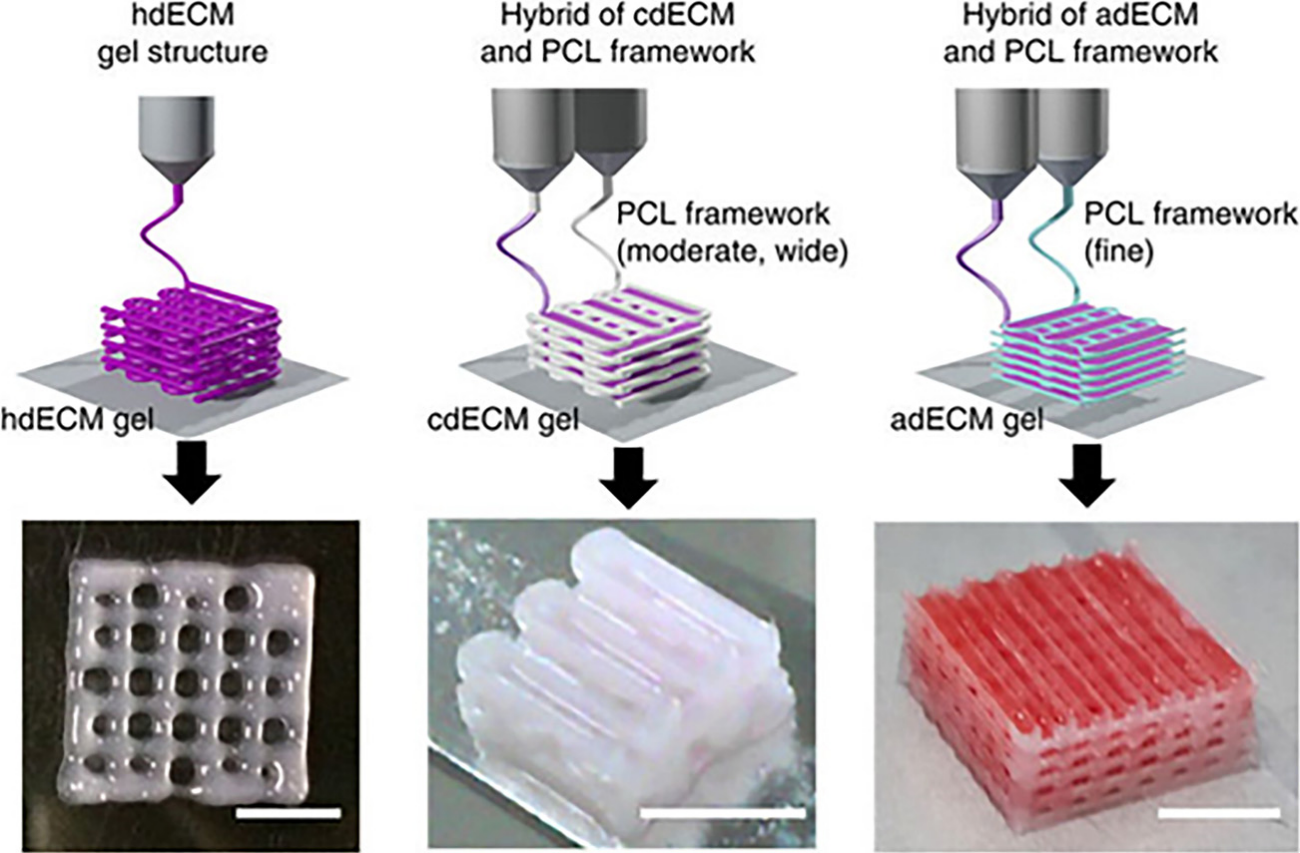
3D printing of bioink. Bioinks made from heart, cartilage, and adipose dECM could be 3D printed either with (cartilage, adipose) or without (heart) PCL framework to create porous scaffolds. Reprinted by permission from Macmillan Publishers Ltd: Nature Communications, Ref. 101, copyright 2014

Pati et al. have demonstrated the fabrication of cell‐seeded bioinks for 3D printing applications.^101^ Cartilage dECM was recellularized with hTMSCs and printed into a porous PCL framework to generate a cartilage tissue scaffold. The authors created wells by first printing multiple layers of a PCL lattice into which a bioink could be deposited (Figure 3).^101^ This process was repeated to create a scaffold several millimeters in height. Seeded hTMSCs exhibited chondrogenic differentiation and showed increased cell viability on the dECM printed scaffold compared to PCL alone.

In addition to cartilage, efforts have been made towards creating bioinks for several other tissue types such as cardiac muscle, skeletal muscle, and liver tissues. Skardal et al. have developed a PEG‐crosslinked, hyaluronic acid/gelatin‐based modular hydrogel system which allows for enhanced control of the biochemical and mechanical properties of the material.^102^ By utilizing two crosslinkers and two separate polymerization steps, the authors were able to fabricate a bioink that has a low enough viscosity to be printed and, following printing, increase the elastic modulus of the scaffold through a secondary polymerization step. dECM from different tissues (e.g.,liver, cardiac, skeletal) have been used with this tunable hydrogel system to formulate bioinks that replicate not only the physical but also the biological properties of the representative tissue.^102^

Utilizing dECM on its own as a bioink for 3D printing is challenging due to its low viscosity and mechanical instability.^101^, ^103^ Bulk hydrogels formed using solubilized dECM only reach stiffnesses similar to or slightly better than that of pure collagen gels^71^ and have very slow gelation times, ranging from 30 minutes to an hour.^104^, ^105^ Although increasing the weight percent of a dECM hydrogel can improve its stiffness,^106^ using such methods for 3D printing is limited as the stiffness of the dECM bioink must be low enough to achieve a viscosity that enables dECM to be extruded through the needle. As such, multiple efforts have been devoted to enhancing the printability of dECM bioinks and the mechanical stability of printed scaffolds by combining them with secondary polymer frameworks,^101^ mixing them with synthetic polymers,^102^ and using crosslinkers.^103^ For instance, adding 0.02% vitamin B2 to a 2% heart dECM bioink and UV‐crosslinking the construct after each layer allowed the final construct to reach a compressive modulus of 15.74 kPa, compared to that of a non UV‐crosslinked construct at 0.18 kPa.^103^ For applications such as bone tissue where much higher moduli are required, dECM bioinks can be combined with 3D printed porous PCL scaffolds, which can reach compressive moduli in the MPa range.^107^ In addition, there have also been efforts to develop shear‐thinning hydrogels that can flow through the needle and retain their shape after they have been printed.^108^, ^109^, ^110^ Such approaches may be translated into the printing of dECM to improve its printability.

### 3.5 Decellularized ECM particles

Rather than being used as a standalone scaffold, dECM can be milled into particles that contain ECM components inherent to the tissue type and can provide binding sites for cells. These particles can then be combined with nonbioactive synthetic or biological scaffolds to form a composite scaffold that have tissue‐specific bioactivity. This flexibility allows dECM particles to be incorporated into various types of scaffolds, such as (a) hydrogels, (b) electrospun scaffolds, and (c) 3D‐printed scaffolds.

#### 3.5.1 Hydrogels

Townsend et al. combined HA, hyaluronic acid, and DCC dECM particles in PBS to fabricate a colloidal suspension with a paste‐like consistency that was shown to enhance bone regeneration in vivo.^111^ Cartilage dECM particles have also been incorporated in type I collagen gels and have shown to enhance chondrogenic gene expression from MSCs encapsulated within the gels. The effect was amplified when the constructs were cultured in chondrogenic media containing transforming growth factor beta‐3 (TGF‐β3).^112^ To enhance the retention of dECM particles inside a hydrogel, Beachley et al. chemically crosslinked dECM particles with modified GAGs (chondroitin sulfate, hyaluronic acid) via carbodiimide chemistry.^113^ Constructs containing bone dECM particles enhanced in vivo bone formation when compared to hydrogels that did not contain dECM particles.

#### 3.5.2 Electrospun scaffolds

**Figure 4:**
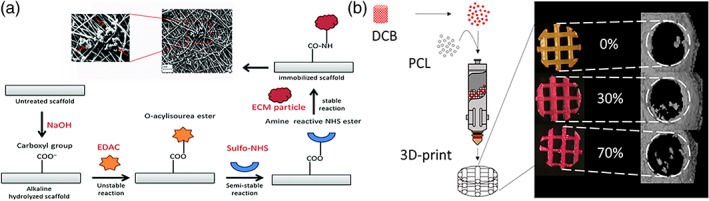
Specific applications for utilizing dECM as particles. (a) Incorporating dECM on electrospun scaffold. NHS groups are introduced on the surface of electrospun poly(hydroxyalkanoate) scaffold via carbodiimide chemistry, which then acts as a binding site for dECM particles. (Insert) Scanning electron microscopy shows the presence of dECM particles (red arrows) on the surface of the scaffold. (b) Incorporating dECM for 3D printing. Decellularized bone ECM particles are mixed with PCL which results in a hybrid scaffold that is printable up to 70% bone dECM by mass. (a) Reproduced in part from Ref. 11 with permission from The Royal Society of Chemistry. (b) Reprinted with permission from Ref. 121. Copyright 2016 American Chemical Society

dECM particles can be incorporated into electrospun scaffolds either during^27^ or after electrospinning.^114^ Garrigues et al. dissolved dECM particles in isopropanol and added PCL to increase the solution's viscosity for electrospinning.^27^ The resulting dECM‐containing electrospun scaffolds showed higher sGAG content and increased collagen synthesis activity from seeded ADSCs compared to PCL scaffolds. Masaeli et al. captured dECM particles on the surface of electrospun polyhydroxyalkanoate via carbodiimide chemistry (Figure 4a).^114^ Human chondrocytes and ADSCs seeded on dECM‐modified scaffolds expressed higher levels of chondrogenic genes compared to cells seeded on unmodified scaffolds*,* indicating that the addition of dECM particles could prevent dedifferentiation of chondrocytes and guide the chondrogenic differentiation of ADSCs. Such treatments improved chondrogenic activity from seeded chondrocytes and MSCs without impacting the scaffold's mechanical properties.

#### 3.5.3 3D‐printed scaffolds

3D printing of PCL fibers is a relatively well‐characterized method used to create porous scaffolds for tissue engineering applications with specific focus on bone tissue regeneration.^107^, ^115^, ^116^, ^117^ To enhance tissue growth, PCL has been combined with bioactive components or minerals such as HA for printing,^118^, ^119^, ^120^ and recently, there have been efforts to incorporate dECM particles to improve bioactivity of PCL scaffolds.^121^, ^122^ Hung et al. have successfully printed porous dECM‐PCL hybrid scaffolds with varying amounts of bone dECM particles and reported that ADSCs better adhered to a printed fiber surface and expressed higher levels of osteogenic gene markers compared to PCL alone (Figure 4b).^121^ dECM particle‐containing scaffolds also generated greater bone volume in vivo. In a similar study, Nyberg et al. performed an in vitro study to assess the behavior of human ADSCs seeded on 3D‐printed PCL scaffolds incorporating bone dECM particles.^122^ The authors observed enhanced bone deposition and increased expression of osteogenic genes such as osteonectin compared to PCL scaffolds containing nonbiological components commonly used to promote osteogenesis such as HA or TCP. This change in cellular response was attributed to the presence of collagen, as well as the natural apatite structure in dECM particles.^122^

#### 3.5.4 Particle aggregates

Cell‐seeded dECM particles can be delivered into the defect site as a particle aggregate.^123^, ^124^ Yin et al. generated dECM particles from goat articular cartilage. These dECM particles were then seeded with MSCs and aggregated in a rotary cell culture system.^123^ Teng et al. cultured cartilage‐like tissue by culturing chondrocyte spheroids in suspension in vitro, which were subsequently decellularized and milled into particles.^124^ In both cases, dECM particles not only induced chondrogenic behavior from seeded MSCs in vitro, but the MSC/particle aggregate also promoted osteochondral defect repair in vivo.

## 4 SUMMARY AND FUTURE PERSPECTIVES

Decellularized ECM is a tissue‐derived biomaterial that can be used as a bioactive component for tissue engineering applications. The versatility of dECM allows it to be processed for various applications, from a whole tissue scaffold to a digested solution that could be used as a bioink for 3D printing. Many dECM studies have shown promising results; the addition of dECM frequently exhibited enhanced regenerative capabilities both in vitro and in vivo and guided the differentiation of seeded stem cells along tissue‐specific lineages even without the addition of exogenous growth factors.

There are, however, limitations to the use of dECM in standard clinical treatments. First, there are significantly different reported methods of dECM tissue processing. With individual studies exploring different combinations of decellularization methods and following flexible guidelines, it is difficult to draw conclusions on which method is best for a specific application. Additional guidelines that not only discuss the removal of nuclear material but also the retention of ECM molecules would contribute greatly to standardized decellularization procedures. Balancing the constraints between removing enough cellular material so as not to elicit an immune response while maintaining ECM composition to preserve bioactivity is a challenge that requires further investigation. Furthermore, tissue sources and storage conditions before being processed for decellularization also influence the quality of dECM, resulting in batch‐to‐batch differences even within the same tissue type. Universally accepted quality control measures for tissue sourcing and storage of dECM would prove beneficial in creating a more reliable and repeatable system.

To summarize, utilization of dECM in tissue engineering applications is still in development and a large portion of the current work still focuses on exploring the effects of different decellularization methods on the biochemical composition of dECM. The results from studies with similar applications are oftentimes contradictory partly because these studies rarely follow the same procedures. In addition, variability in tissue processing—from isolating the tissue to decellularizing—results in low reproducibility. Thus, further studies are necessary if the relationship between the decellularization process and the resulting composition of dECM is to be better understood. General decellularization guidelines that could widely be agreed upon by investigators will pave the way for a more standardized field of tissue decellularization. In addition, the development of methods to enhance the physicochemical properties of dECM while harnessing its native regenerative capacities will be the key to providing viable therapeutic applications for bone and cartilage tissue regeneration.
